# Genomic and epigenomic diversity of breast cancer across Western and MENA populations: implications for precision oncology

**DOI:** 10.3389/fonc.2026.1898542

**Published:** 2026-08-27

**Authors:** Saniyah Shaikh, Arshiya Akbar, Hafsah Tajammul Khalifey, Thaabit Raziq, Dina Abu-Saleh, Hibah Alruwaili, Shrouq Almansouri, Itika Arora, Edwin Aroke, Khalid Walid Freij, Mohammad Imran Khan, Ahmed Yaqinuddin

**Affiliations:** 1 College of Medicine, Alfaisal University, Riyadh, Saudi Arabia; 2 Department of Acute, Chronic and Continuing Care, School of Nursing, University of Alabama at Birmingham, Birmingham, AL, United States; 3 King Faisal Specialist Hospital and Research Center, Jeddah, Saudi Arabia

**Keywords:** ancestry, BRCA, breast cancer, epigenomics, MENA, pharmacogenomics, precision oncology, Saudi Arabia

## Abstract

Breast cancer is the most common malignancy in women worldwide and is increasingly recognized as a biologically diverse disease shaped by both molecular and ancestral context. Women from the Middle East and North Africa (MENA) populations, including Saudi Arabia, often present at a younger age and with more aggressive subtypes such as HER2-positive and triple-negative breast cancer (TNBC) compared with Western cohorts. These clinical patterns reflect a distinctive genomic background marked by high consanguinity, founder mutations in key susceptibility genes, and population-specific somatic alterations that are not fully captured in global reference datasets. This review brings together current evidence on somatic, germline, transcriptomic, and epigenomic diversity in breast cancer across Western and MENA populations, with a focus on Saudi cohorts. Drawing on a previously published systematic review of more than 2,500 MENA breast cancer cases, *TP53* accounted for approximately 24% and *PIK3CA* for roughly 10% of curated somatic mutation records pooled across 44 studies (proportions of mutation calls, not per-patient prevalence); in a separate single-center Saudi cohort, only 3.7% of patients underwent BRCA testing, and 37.5% of this clinically selected, testing-referred subgroup carried a pathogenic variant, a figure that should not be read as general-population BRCA prevalence. Variants of uncertain significance exceeded 20% across several regional genomic studies. We summarize conserved driver events, such as recurrent *TP53* and *PIK3CA* mutations, while highlighting regional features, including unique stop-gain and loss-of-function variants, a high copy-number burden, and early-onset disease linked to ancestral architecture. We also discuss emerging data on MENA-specific regulatory signatures, including immune-enriched and basal-myo transcriptomic clusters, CIMP-like methylation patterns, and non-coding RNA networks; these associations are numerically suggestive in available cohorts but have not reached statistical significance in existing studies and warrant validation in larger, dedicated MENA/Saudi cohorts before being considered established determinants of treatment response and resistance. Finally, we examine the clinical implications of this diversity for biomarker development, pharmacogenomics, and access to targeted therapies, and outline practical steps toward ancestry-aware precision oncology in the region.

## 1 Introduction

Breast cancer is the most prevalent cancer among women worldwide and remains a leading cause of mortality. Incidence is rising across both high and low-resource regions (1, 2). Its clinical complexity reflects profound molecular heterogeneity arising from somatic mutations, copy-number variations (CNV), and transcriptomic and epigenomic alterations that influence tumor initiation, progression, and therapy response (3, 4). Microenvironmental pressures, including hypoxia, epithelial-mesenchymal plasticity, and immune infiltration, further shape intertumoral diversity and impact prognosis (5, 6).

Ancestral genomic background critically modulates these molecular features. Evidence indicates that MENA populations, particularly Saudi women, exhibit earlier age at diagnosis and higher prevalence of aggressive subtypes such as HER2-positive and triple-negative breast cancer (TNBC) relative to Western cohorts. These subtypes are associated with poorer prognosis and lower response to standard therapies (7–9). These observations derive from unadjusted comparisons and are confounded by the younger population age structure, variable screening coverage, and differences in referral pathways and stage at diagnosis (§3.2); a genuine ancestral contribution remains plausible, arising from germline architectures, founder mutations and population-specific somatic alterations that, in combination with epigenetic and transcriptomic regulation, may alter therapeutic response and survival outcomes (10, 11).

Despite the global expansion of genomic profiling, cancer genomics research remains dominated by individuals of European ancestry, who comprise nearly 80% of GWAS participants despite representing only 16% of the global population, whereas MENA populations remain underrepresented in genomic studies. This underrepresentation contributes to higher frequencies of variants of uncertain significance, limits the detection of ancestry-specific drivers, and reduces the clinical utility of predictive models calibrated to European-ancestry cohorts (12–14). For clarity, we use "Western" to refer collectively to studies and reference datasets predominantly composed of European-ancestry participants (e.g., TCGA, METABRIC), and "European" specifically when a cited study restricts its cohort to self-reported European ancestry. “MENA” refers broadly to the Middle East and North Africa; where findings are specific to Saudi Arabia or the Gulf Cooperation Council, this is stated explicitly rather than generalized to the full MENA region. “Arab” is used only where a cited source applied that designation to its own participants, and is not used as a synonym for MENA or Saudi.

This review synthesizes current knowledge of genomic and epigenomic diversity in breast cancer across Western and MENA populations with a focus on Saudi Arabia, highlighting population-specific patterns, underrepresentation in global datasets, and implications for precision oncology (15, 16).

## 2 Methodology

This manuscript was prepared as a structured narrative review. It does not constitute a systematic review or meta-analysis, and was not conducted according to PRISMA methodology. Pooled frequency estimates cited throughout, including the MENA mutation prevalence figures summarized in the Abstract, are drawn from previously published systematic reviews and primary studies rather than from a *de novo* systematic search or quantitative synthesis performed here. Relevant literature was identified through searches of PubMed/MEDLINE, Scopus, and Google Scholar for studies published through May 2026. Search terms included combinations of “breast cancer”, “MENA”, “Middle East”, “Saudi Arabia”, “genomics”, “somatic mutations”, “germline variants”, “BRCA”, “epigenomics”, “DNA methylation”, “transcriptomics”, “pharmacogenomics”, “ancestry”, and “precision oncology”.

Additional relevant publications were identified through reference screening of key articles and reviews. Searches were restricted to English-language publications; no restriction was applied by publication date beyond the cutoff described above. Eligible sources included peer-reviewed original research articles, systematic reviews, meta-analyses, population-based genomic studies, and major cancer genomics resources reporting molecular, genomic, transcriptomic, epigenomic, or pharmacogenomic findings relevant to breast cancer. Studies involving Saudi Arabian, Gulf Cooperation Council (GCC), Arab, and broader MENA populations were prioritized, while landmark international datasets were included for comparative context.

Publications unrelated to breast cancer, lacking molecular characterization, or providing insufficient methodological detail were excluded. The literature was synthesized narratively and organized into thematic domains encompassing population ancestry, somatic and germline alterations, transcriptomic and epigenomic regulation, pharmacogenomics, and clinical translation. Owing to substantial heterogeneity in study design, sequencing platforms, patient populations, and reported outcomes, a formal meta-analysis was not performed.

## 3 Population context and ancestral architecture in MENA

### 3.1 Genetic ancestry, admixture, and database bias

**Figure 1:**
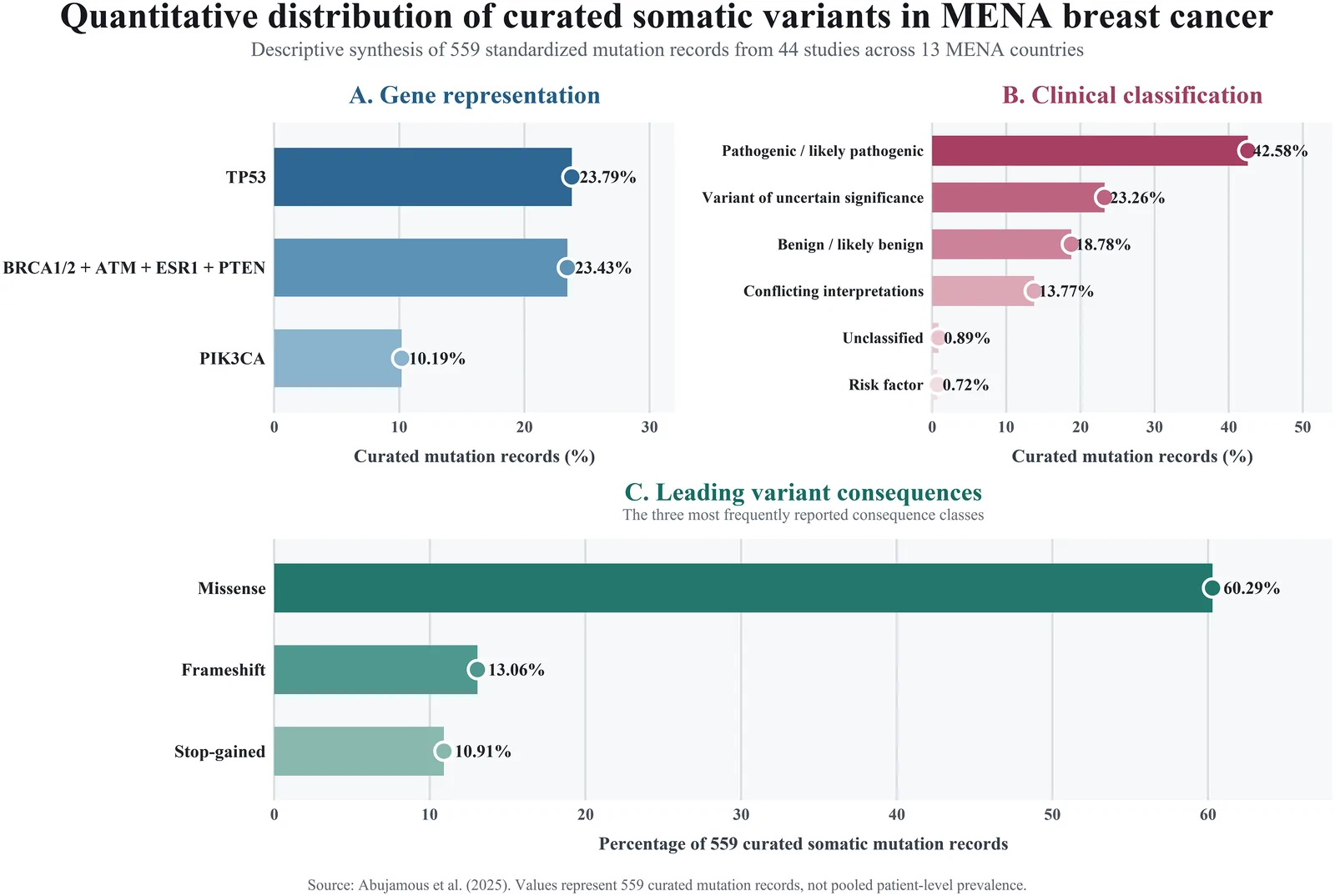
Distribution of curated somatic mutation calls in MENA breast cancer. **(A)** Relative representation of *TP53*, *PIK3CA*, and the combined *BRCA1/2*, *ATM*, *ESR1*, and *PTEN* group among 559 mutation calls curated from 44 studies across 13 MENA countries. **(B)** Clinical classification of the same 559 calls into six categories, summing to 100%. **(C)** The three most frequently reported variant-consequence classes among these calls. Values represent proportions of curated mutation calls rather than per-patient prevalence and are drawn from a narrative synthesis (12); as no meta-analysis was performed in the source dataset, no pooled estimates or confidence intervals are available or shown.

Populations in the MENA region are genetically heterogeneous, shaped by admixture between ancient Levantine, Arabian Peninsula, and North African lineages. Consanguinity and endogamy, prevalent in Saudi Arabia and surrounding Gulf countries, elevate homozygosity and increase the frequency of pathogenic alleles, including founder mutations in key breast cancer susceptibility genes (*BRCA1*/2, *TP53*, *ATM*, *PALB2*) (17–19). Pronounced founder effects generate recurrent pathogenic variants shared among unrelated individuals, which influence age at diagnosis, tumor aggressiveness, and hereditary cancer risk profiles (19–21). As illustrated in **Figure 1**.

**Figure 2:**
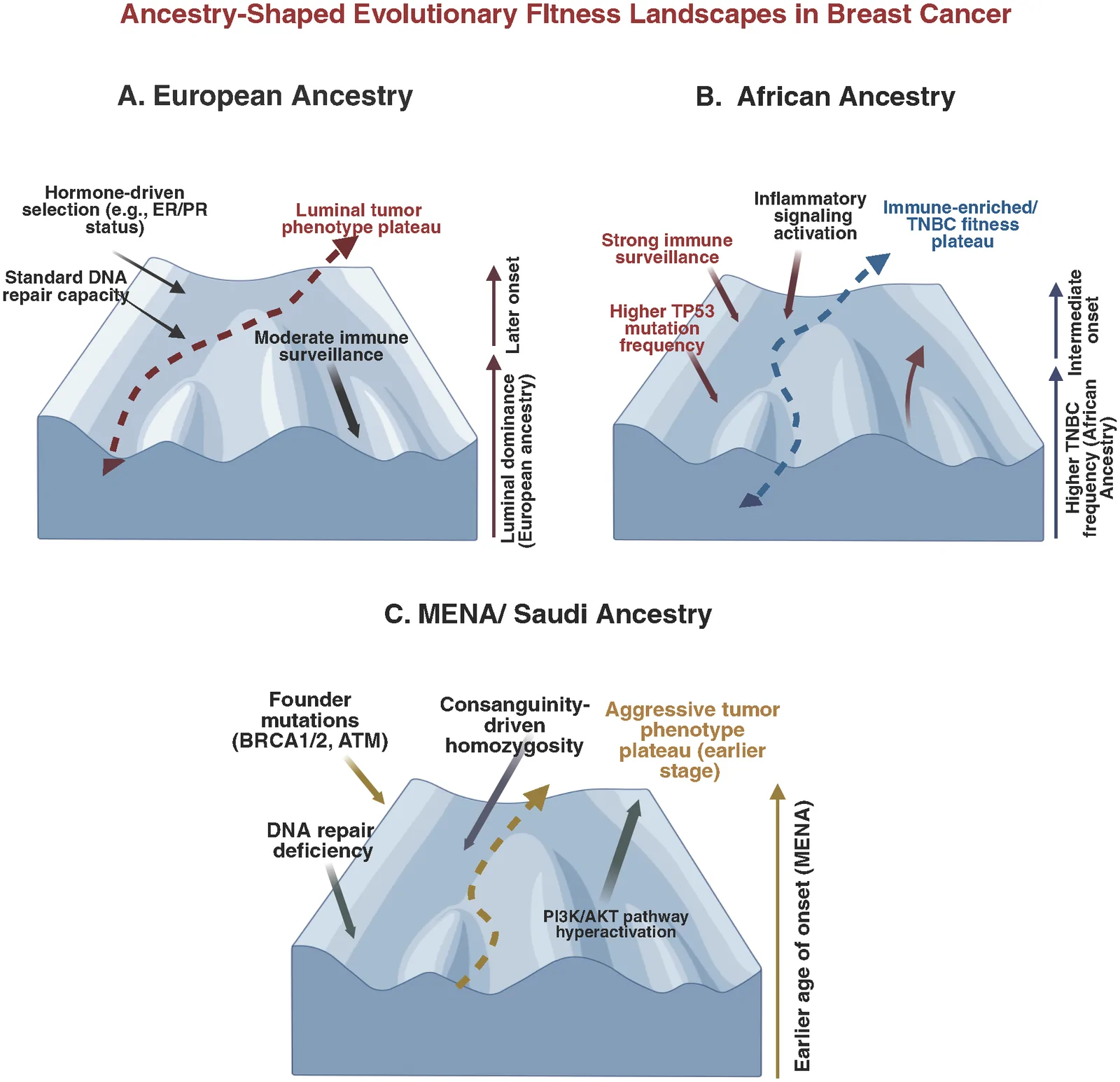
Ancestry-associated conceptual fitness landscapes in breast cancer (hypothesis-generating model). Schematic fitness landscapes illustrating the hypothesis that ancestry-associated genomic architecture may differentially shape breast cancer evolutionary trajectories. **(A)** European ancestry: a proposed fitness landscape favoring luminal phenotypes through hormone-driven selection and moderate immune surveillance. **(B)** African ancestry: a proposed immune-enriched/triple-negative breast cancer (TNBC) fitness plateau associated with stronger immune surveillance, inflammatory signaling, and a higher frequency of *TP53* mutations. **(C)** MENA/Saudi ancestry: a proposed landscape characterized by founder mutations, including *BRCA1/2* and *ATM*, consanguinity-associated homozygosity, DNA-repair deficiency, and PI3K/AKT pathway activation, potentially converging on more aggressive tumor phenotypes and earlier age and stage of onset. These landscapes are schematic, illustrative, and not drawn to scale; they are not derived from ancestry-stratified evolutionary modeling data and do not establish causal relationships between ancestry and tumor phenotype. Rather, the model provides a conceptual framework for generating testable hypotheses for future ancestry-stratified genomic and functional studies.

**Figure 2** Ancestry-shaped evolutionary fitness landscapes in breast cancer. Fitness landscape models illustrate how ancestral architecture shapes tumor evolution. European ancestry (A) favors luminal phenotypes via hormone-driven selection and moderate immune surveillance. African ancestry (B) drives an immune-enriched/TNBC fitness plateau through strong immune surveillance and inflammatory signaling, with a higher frequency of *TP53* mutations. MENA/Saudi ancestry and (C) is defined by founder mutations (*BRCA1*/2, *ATM*), consanguinity-driven homozygosity, DNA repair deficiency, and PI3K/AKT hyperactivation, converging on an aggressive tumor phenotype, with an earlier stage and age of onset. The landscapes are schematic and illustrative, intended as conceptual heuristics rather than data-derived models, and are not drawn to scale.

Despite this genetic complexity, MENA populations are severely underrepresented in genomic reference datasets such as TCGA, gnomAD, and the GWAS catalog, where fewer than 2% of GWAS participants are drawn from Africa or the Middle East (3). These biases limit the identification of population-specific somatic drivers, mutational signatures, and germline variants, leading to high rates of variants of unknown significance and constraining the effectiveness of precision oncology tools designed for European cohorts (22, 23). Population-specific genomic studies are therefore critical for refining biomarker discovery, improving variant interpretation, and guiding ancestry-aware clinical decision-making in MENA populations (24, 25).

### 3.2 Environmental and lifestyle modifiers

Genomic susceptibility interacts with environmental and lifestyle factors to shape breast cancer risk and tumor phenotype. A case-control study from Saudi Arabia (135 cases, 270 controls) found that body mass index, parity, marital status, and menopausal status were significant independent predictors of breast cancer risk on multivariate logistic regression; women with a BMI >30 kg/m² (42.3% of cases vs. 30.8% of controls) and fewer children were significantly overrepresented among cases, whereas breastfeeding history did not differ significantly between groups in this cohort (26). A population attributable risk analysis (PAR) estimated that physical inactivity accounted for the largest proportion of potentially preventable breast cancer burden among Saudi women (PAR 23.1%), followed by current oral contraceptive use (14.5%), postmenopausal obesity (8.9%), hormone replacement therapy (5.2%), passive smoking (4.5%), age at first birth ≥35 years (0.7%), and tobacco smoking (0.5%) (27). Dietary exposures may further influence risk across the MENA region, with healthy dietary patterns associated with reduced risk and inflammatory or unhealthy dietary patterns associated with increased risk (28).

Access to screening programs is variable across the region, affecting stage at diagnosis and clinical outcomes, while dietary and lifestyle changes modulate the manifestation of inherited cancer risk. These environmental and lifestyle factors interact mechanistically with ancestral genomic variants, shaping both the mutational landscape and epigenomic regulation (29, 30). Integrated models that consider ancestry, environment, and lifestyle are therefore essential for elucidating population-specific oncogenic mechanisms and informing prevention strategies in MENA populations.

## 4 The genomic landscape: somatic and germline alterations

Breast cancer is now characterized by a complex genomic landscape that combines inherited germline alterations and acquired somatic mutations, rather than by basic histology alone. Although global studies such as TCGA have laid the foundation for understanding these mutation patterns, recent work indicates that the effects of somatic drivers and germline predisposition are largely determined by population ancestral origins (31, 32). Understanding this integrated genomic architecture is essential for moving beyond Western-centric models toward a truly global precision oncology framework.

### 4.1 Core somatic driver mutations across populations

A conserved set of driver alterations characterizes the genomic landscape of breast cancer, although their distribution and biological impact vary considerably among molecular subtypes and populations.

The original TCGA breast cancer analysis and subsequent large-scale resequencing efforts have converged on *TP53, PIK3CA*, and *GATA3* as among the most recurrently altered genes across breast cancer as a whole (7, 8, 33).

Both TCGA and other Western datasets demonstrate a stable, subtype-specific mutation signature. Basal-like and HER2-enriched subtypes harbor abundant *TP53* mutations resulting from *TP53* loss and marked genomic instability. Luminal subtypes predominantly harbor *PIK3CA* and *GATA3*/*ESR1* mutations, inducing estrogen-driven growth (7, 34). Recurrent chromosomal gains in 1q, 11q (*CCND1)*, and 17q (*ERBB2*) define clinical behaviors (35–37). High copy-number variation burden frequently has a more substantial predictive effect than point mutation burden alone, closely linked to higher tumor stage and poor clinical outcome (10, 38).

### 4.2 Population-specific somatic variation in MENA/Saudi cohorts

The following somatic mutation frequencies were derived from tumor-only sequencing and reflect acquired, non-inherited alterations. MENA and Saudi breast tumors have unique mutational regimes, including population-specific variations that are uncommon or nonexistent in Western cohorts (39, 40). Personalized sequencing in

**Figure 3:**
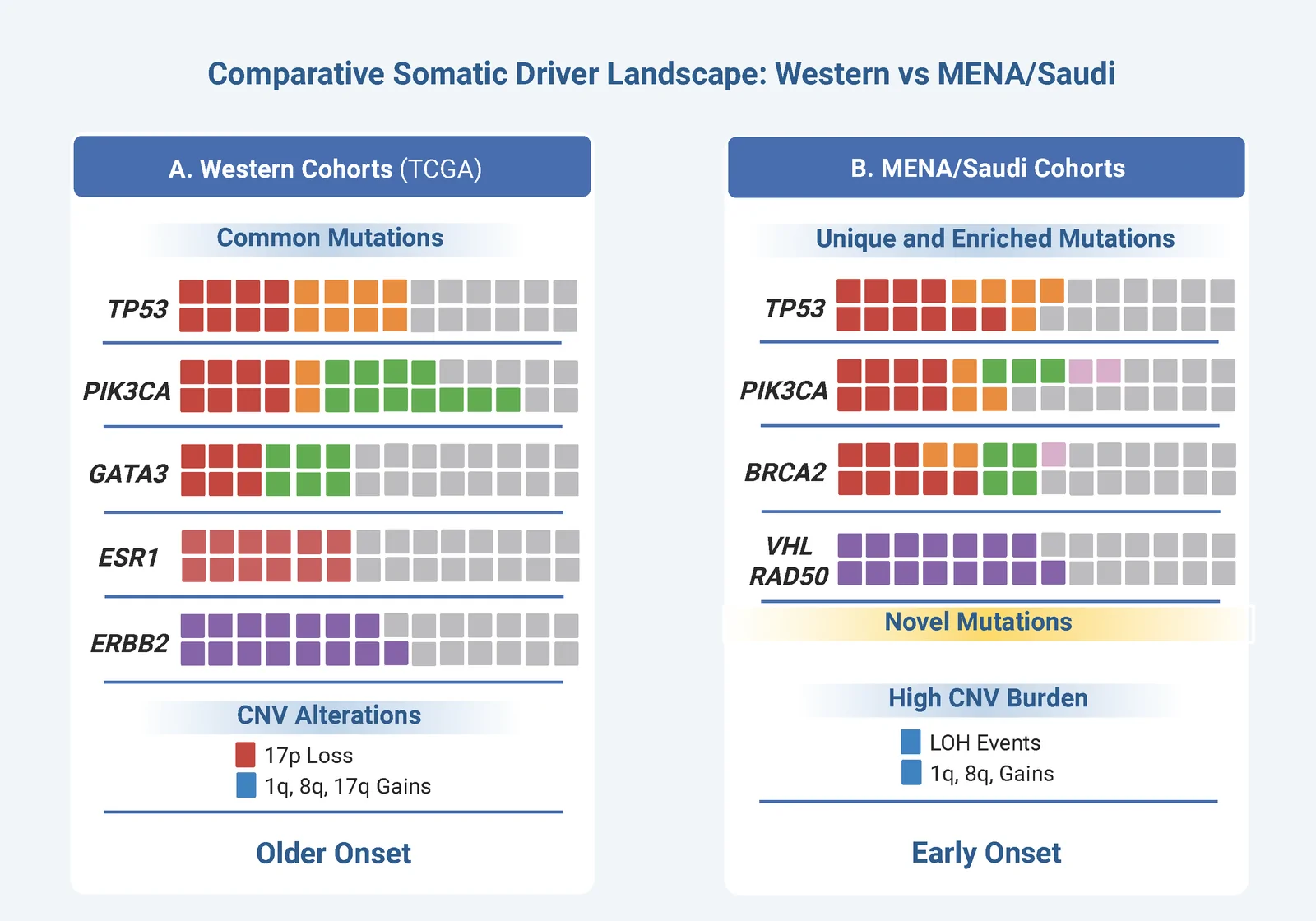
Comparative somatic driver landscape in Western (TCGA) versus MENA/Saudi breast cancer cohorts. Western cohorts **(A)** are characterized by *TP53*, *PIK3CA*, *GATA3*, *ESR1*, and *ERBB2* mutations with 17p loss and 1q/8q/17q gains, and later onset. The Saudi cohorts **(B)** share *TP53* and *PIK3CA* alterations but show *BRCA2* enrichment, a distinct novel mutation cluster absent from Western databases, high LOH-driven CNV burden, and earlier age of onset. The oncoprint-style panels are schematic and illustrative, intended to convey relative mutation patterns rather than precise frequencies, and are not drawn to scale; copy-number estimates for Saudi cohorts in particular remain provisional pending dedicated population-level profiling. (The Western and Saudi panels are derived from separate, non-matched cohorts using different sequencing platforms and variant-calling pipelines (see Methods); they are presented side-by-side for descriptive comparison only and should not be interpreted as a matched or statistically comparable frequency plot).

Saudi patients showed loss of heterozygosity (LOH) in genes including *VHL, GPC3, RAD50, BUB1*, and *SETBP1*, as well as completely disabled tumor-suppressor genes and altered RNA processing, influenced by ancestry and sociocultural history (40, 41). Direct large-scale CNV profiling of Saudi cohorts remains limited, and available data should be considered provisional pending dedicated population-level studies. The limitations of Western reference databases were further demonstrated by pathogenic variants in Saudi patients; including stop-gain mutations in *TP53, VHL*, and *BRCA2*, misclassified as variants of uncertain significance (42). As shown in **Figure 3**.

The somatic landscape of Saudi patients shows both population-differential frequencies and population-unique variants: *PIK3CA* (12.9%), *BRCA2* (11.7%), and *TP53* (6.0%) are known breast cancer driver genes occurring at gene-level frequencies that differ from Western benchmarks, whereas the accompanying 46 novel predicted-pathogenic variants across 22 genes appear to be genuinely absent from major Western reference datasets (37, 43). Aggressive clinicopathological features, such as high Ki-67 levels reported in over 70% of cases in some regional series, suggest a distinct tumor biology in the area (44, 45).

### 4.3 Germline variants and founder effects in MENA

In contrast to the somatic alterations described above, this section addresses inherited, germline predisposition variants confirmed through blood or normal-tissue testing.

Familial breast cancer is predominantly associated with deleterious germline mutations in *BRCA1, BRCA2, PALB2, ATM*, and *TP53*; however, mutation profiles and penetrance vary considerably in MENA populations compared to European cohorts (20). In Saudi Arabia, *BRCA1* pathogenic variants are common, especially in ovarian cancer patients, consistent with the founder effect (35). While some reports show reduced *BRCA1*/2 prevalence compared to Egypt or Qatar (46), the high level of consanguinity and earlier age at diagnosis (at least 10 years younger than in Western populations) indicate a strong inherited component (9, 20).

Elevated levels of consanguinity and endogamy have increased homozygosity for deleterious alleles and amplified population-specific mutations (18). Proof of founder effect is the surprisingly high frequency of recurrent *BRCA1* and *BRCA2* variants (19). Importantly, common haplotypes and frequent LOH occurrences across unrelated individuals provide evidence that ancestral founder alleles spread within extended families (21, 47).

The founder-mutation spectrum in MENA populations includes variants often absent from major reference panels; for example, stop-gain mutations in *TP53, VHL*, and *BRCA2* identified in Saudi patients have been misclassified as variants of uncertain significance on panels calibrated to European-ancestry reference data (42, 48). This indicates that variant curation and reference-database composition, rather than panel technology broadly, require ancestry-aware recalibration.

Therefore, the adoption of broader sequencing methods and ancestry-aware genetic tests is necessary to move beyond “one-size-fits-all” treatments (4, 49). **Table 1** summarizes the reported somatic and germline mutation landscape across MENA/Saudi and Western cohorts.

**Table 1:** Reported somatic and germline mutation atlas for MENA/Saudi breast cancer.

Gene/pathway	Variant class	MENA/Saudi finding	Western benchmark	level	Ref.
Somatic mutations					
*TP53*	Missense/stop-gain/frameshift	Most frequently in MENA meta-analysis: 23.79% pooled (12). Saudi NGS panel: 6.0% gene-level (37). Stop-gain and LOF variants enriched; linked to basal-like/TNBC.	Basal-like 80–90%; luminal 10–30%	Somatic	(12, 37)
*PIK3CA*	Missense hotspot (H1047R, E542K, E545K)	10.19% pooled MENA meta-analysis (12); 12.9% gene-level Saudi panel; H1047R recurrent at 2.6% (37). All 11 alpelisib-predictive hotspots are present across MENA.	Most common in luminal/HER2+ (~30–40% in ER+ disease).	Somatic	(12, 37)
*BRCA2* somatic	Frameshift/missense; I2285V recurrent	11.7% gene-level Saudi panel is the highest after *PIK3CA* (37). *BRCA2* independently predicted poorer OS on Cox multivariate analysis.	Somatic *BRCA2* ~5–10% pan-cancer.	Somatic	(37)
*BRCA1* somatic	Frameshift/missense; N550H recurrent (1.15%)	10.2% gene-level Saudi panel (37). N550H confirmed recurrent. Reduced survival in *BRCA1*-mutant patients.	Somatic *BRCA1* ~5% pan-cancer.	Somatic	(37)
*MSH2/MLH1/ PMS2/MSH6*	Pathogenic missense/truncation	Saudi panel: MSH2 3.8%, PMS2 3.8%, MLH1 3.4%, MSH6 3.0% (37). Signals dMMR/MSI-high subgroup.	MMR somatic ~2–5% BC globally.	Somatic	(37)
Germline variants and founder effects					
MENA multi-gene panel	*BRCA1*/2, ATM, ESR1, PTEN + others	44 studies, 13 countries, >2,500 patients (12): VUS 23.26% (12); P/LP 43%; missense 60.29%; all 11 *PIK3CA* alpelisib hotspots present.	VUS ~10–15% in European-ancestry cohorts.	Pooled literature	(12)
*PALB2* c.3425delT	Frameshift; Saudi founder	Recurrent in ~1/3 of *PALB2*-positive patients (n=918 BC+OC cohort) (19). Additional non-BRCA variants: MLH1, MLH3, MUTYH, MSH2, MSH6.	No equivalent Saudi founder in Western panels.	Germline	(19)
*BRCA1* germline	Pathogenic; 3 Saudi founder variants	11% of 310 high-risk Saudi BC patients (50). Three founders: c.4136_4137delCT, c.5530delC, c.4524G>A. TNBC in 86% of BRCA-mutant cases (50).	~5–10% high-risk Western cohorts. Ashkenazi founders differ completely.	Germline	(50)

Gene-level frequencies [Barakeh 2021 (37)] reflect proportions across 263 total mutations in a 51-gene NGS panel, not per-patient carrier rates. Pooled frequencies [Abujamous 2025 (12)] are based on 44 studies involving >2,500 patients across 13 countries. BC, breast cancer; TNBC, triple-negative BC; LOH, loss of heterozygosity; VUS, variant of uncertain significance; OS, overall survival; LFS, Li-Fraumeni Syndrome; P/LP, pathogenic/likely pathogenic; dMMR, deficient mismatch repair; WES, whole-exome sequencing.

## 5 Epigenomic and transcriptomic regulatory diversity

The molecular structure of breast cancer encompasses not only alterations in DNA sequences but also a complex regulatory framework influenced by gene expression patterns and epigenetic modifications (51). There is now growing evidence that the regulatory network (transcriptome, methylome, and non-coding RNA) varies across populations. In MENA and Saudi cohorts, separate regulatory signatures may affect tumor growth, immune infiltration, and drug resistance compared to what is documented in Western cohorts (40, 52).

### 5.1 Transcriptomic differences across ancestries

Beyond simple DNA changes, transcriptomic research has identified ancestry-specific molecular clusters that impact malignant behavior. The ‘basal-myo’ transcriptomic cluster and a specific ‘immune enriched’ hallmark have been observed at numerically higher frequencies in Arab ancestry cohorts, though this trend did not reach statistical significance (44) and requires validation in larger dedicated Arab cohorts. These transcriptomic profiles are associated with aggressive clinical characteristics, including higher-stage tumors and tumors with Ki-67>70% in Saudi populations (45).

Differential expression analysis reveals the activation of particular oncogenic pathways in MENA cohorts. Pathways such as PI3K/AKT/mTOR, AMPK, and epithelial-mesenchymal transition (EMT) signatures appear to be active depending on genetic differences and microenvironmental pressures (3, 4). Moreover, TNBC cases in Middle Eastern women often exhibit a “basal-like immune-suppressed” (BLIS) transcriptomic subtype, which is associated with the most unfavorable prognosis and reduced CD8+ T-cell infiltration relative to Western TNBC populations (44).

### 5.2 DNA methylation and CIMP-like patterns

DNA methylation has been the most characterized epigenetic modification involved in silencing tumor suppressors and activating oncogenic programs in Saudi breast cancer (51, 53). Saudi Arabia has shown signs of hypermethylation in the silencing of genes linked to breast cancer, despite the lack of direct large-scale epigenomic comparisons. These patterns have been proposed, by analogy with other TNBC-enriched populations, to resemble the “CpG Island Methylator Phenotype” (CIMP); however, this remains a working hypothesis in Saudi cohorts pending direct large-scale methylome studies (10). The methylation status of key genes such as *BRCA1* and *MGMT* clearly varies across populations. Even in the absence of germline mutations, epigenetic silencing by promoter hypermethylation can produce “BRCAness” in Saudi cohorts, leading to homologous recombination deficiency (HRD) (8). The implications are that, across the population, epigenetic regulation can vary with genetic background and environmental exposures (10). This reversibility is mechanistically relevant to the MENA context. Experimental evidence further indicates that dietary and bioactive compounds can modulate key epigenetic mechanisms, including DNA methylation and histone modifications, thereby influencing the expression of tumor-suppressor genes and pathways involved in breast cancer progression (51). Given the regional predominance of early-onset and triple-negative disease, these pathways identify a plausible, modifiable epigenetic axis worth testing directly in MENA cohorts rather than extrapolating from Western data.

### 5.3 Non-coding RNA and chromatin remodeling

The non-coding transcriptome, including microRNAs (miRNAs) and long non-coding RNAs (lncRNAs), provides an additional layer of ethnicity-specific regulation. Regional investigations have revealed distinct miRNA signatures that regulate cell-cycle control and signaling pathways, which are frequently affected by ancestry-specific variations (40). However, significant gaps remain in our understanding of lncRNA expression and histone modification landscapes within MENA populations (12, 40, 51). Circulating miRNAs are increasingly recognized as dynamic epigenetic biomarkers that respond to environmental and dietary exposures, highlighting the importance of interpreting ancestry-specific miRNA signatures within the context of potentially modifiable environmental influences in MENA populations (28, 51).

In breast cancer, both chromatin modifiers *KMT2C* and *ATRX* appear to be frequently mutated. Early evidence from non-MENA cohorts indicates that differences in the SWI/SNF complex can alter chromatin accessibility at ER-binding regions; whether this mechanism contributes to the reportedly higher frequency of endocrine resistance in Middle Eastern luminal B cases remains untested directly and should be considered hypothesis-generating pending regional functional studies (12). **Table 2** summarizes the ancestry-stratified regulatory landscape across transcriptomics, methylation, and non-coding RNA layers.

**Table 2:** Ancestry-stratified regulatory landscape: transcriptomics, methylation, and non-coding RNA in MENA breast cancer.

Layer	Population/cohort	Method	Key finding (Reported)	Clinical implication	Evidence basis	Ref.
Transcriptomics						
Transcriptome	ArA, AA, EA, TCGA + RA-QA Qatar cohort	TDA-based PAM50; SNP ancestry	basal-myo: ArA 25.0% *vs* EA 16.5% (trend, *p*= n.s.); AA 38.0% vs EA 16.5% (*p* = 1.30×10^-^¹^0^). AMPK HR: AA 2.79 *vs* EA 0.34 (44).	The Arab BC cohort may share basal-myo enrichment with African ancestry; prospective validation in larger Arab cohorts is required before clinical translation. Ancestry-aware subtyping is required before AMPK-targeted therapy	Extrapolated	(44)
Transcriptome	Saudi Arabia BC cohort	Microarray profiling	Altered adiponectin and *FABP4* expression; lipid metabolism pathways differentially activated (52).	*FABP4* as a Saudi-specific biomarker or therapeutic target.	Direct regional	(52)
DNA methylation/epigenomics						
DNA Methylation	Saudi Arabia, TNBC/basal-like	CpG island profiling; *BRCA1*/MGMT promoter methylation	CIMP-like hypermethylation in Saudi TNBC. *BRCA1* promoter methylation creates epigenetic BRCAness/HRD in germline *BRCA1* wild-type patients (10).	Epigenetic BRCAness → HRD → potential PARPi sensitivity without germline mutation.	Extrapolated	(10)
Non-coding RNA and chromatin remodeling						
Chromatin	Saudi Arabia / MENA — emerging data	*KMT2C*/*ATRX* somatic profiling; SWI/SNF analysis	*KMT2C* and *ATRX* are frequently mutated. SWI/SNF differences alter ER-binding chromatin accessibility — proposed mechanism for endocrine resistance in luminal B (12).	Chromatin remodeling may account for differential responses to endocrine therapy in MENA luminal-B BC.	Extrapolated	(12)
Somatic Mutations (DNA)	MENA cohorts (SA, Qatar, Lebanon)	Somatic mutation profiling, driver gene prediction, and therapeutic actionability mapping (OncoKB)	Identified *PIK3CA* and *TP53* as dominant somatic drivers across MENA cohorts, revealing a high enrichment of actionable alterations within the PI3K/AKT/mTOR and RTK/RAS signaling pathways.	A regional miRNA catalog is needed for the development of MENA-relevant biomarkers.	Direct regional	(40)

ArA, Arab ancestry; AA, African ancestry; EA, European ancestry; CIMP, CpG island methylator phenotype; HRD, homologous recombination deficiency; EMT, epithelial-mesenchymal transition; FABP4, fatty acid binding protein 4; AMPK, AMP-activated protein kinase; IHC, immunohistochemistry; TDA, topological data analysis.

### 5.4 Integrated multi-omics models

To comprehensively explain the biological trajectory of breast cancer in the MENA region, there is a clear need to advance toward integrated multi-omics models that reconstruct regulatory networks across the genome, epigenome, and proteome. Current research underscores the shortcomings of a “one-size-fits-all” methodology, as conventional therapy is not effective for all patients due to rapid resistance and population-specific mutations (12, 40, 49).

**Figure 4:**
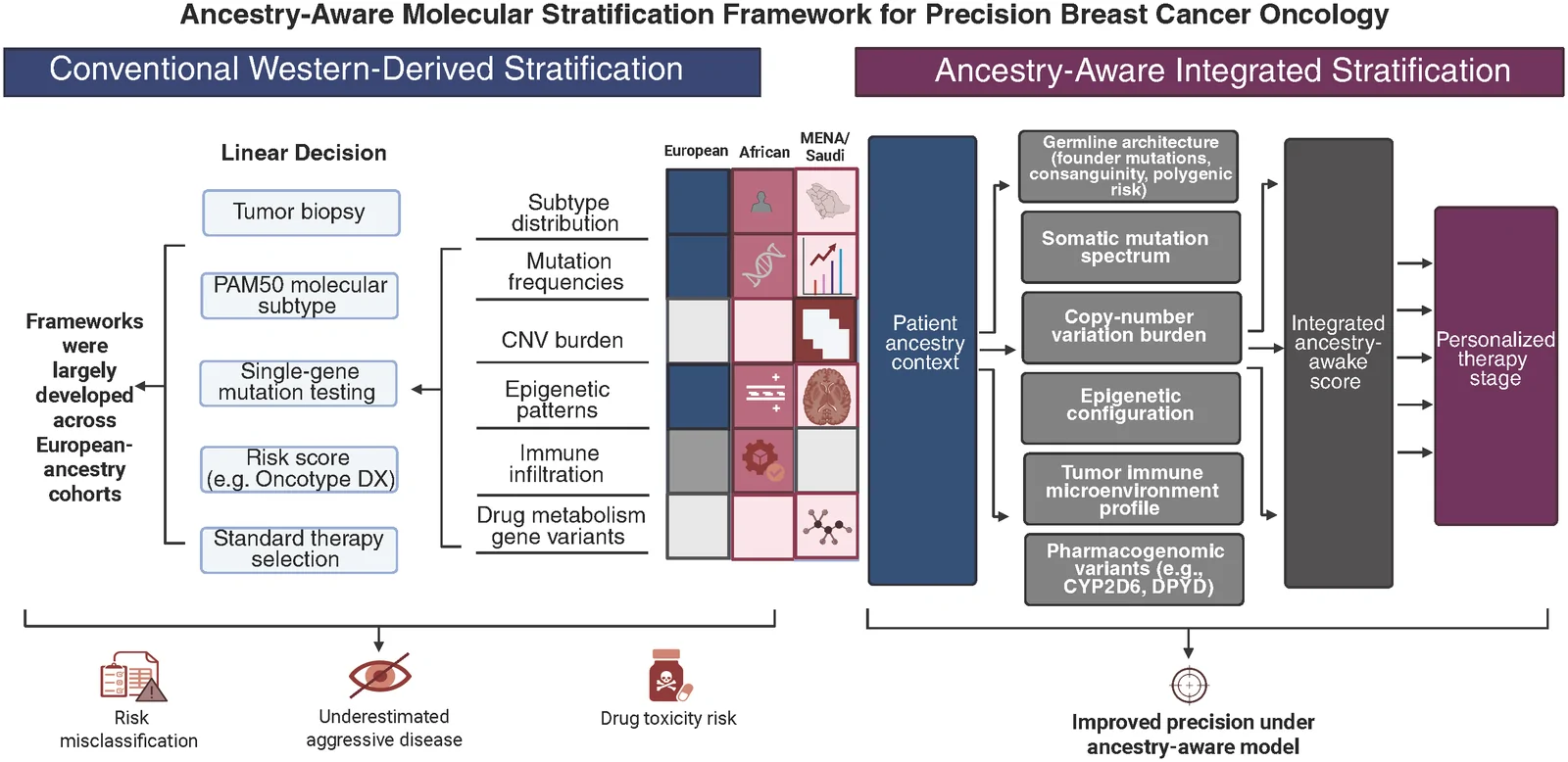
Ancestry-aware molecular stratification framework for precision breast cancer oncology. Conventional Western-derived stratification (left) relies on a linear pipeline, PAM50 subtyping, single-gene testing, and risk scores (e.g., Oncotype DX), developed predominantly on European-ancestry cohorts, leading to risk misclassification, underestimated aggressive disease, and drug toxicity in non-European patients. The proposed ancestry-aware framework (right) integrates six molecular layers, germline architecture, somatic mutation spectrum, CNV burden, epigenetic configuration, tumor immune microenvironment, and pharmacogenomic variants (*CYP2D6*, *DPYD*), weighted by patient ancestry context to generate an integrated score guiding personalized therapy. This framework is proposed rather than validated: its component weights have not been derived, no integrated score has been constructed, and its performance has not been evaluated against existing stratification tools.

These analyses should be coupled with single-cell analyses and proteogenomics to characterize differences between ancestral groups regarding intratumoral heterogeneity and the tumor microenvironment (5). Integrating transcriptomic data with spatial proteomics will allow for a better understanding of how the Saudi “Immune-Enriched” signature translates into actual protein expression and clinical response to immunotherapy (44). Rather than modeling directly on Western data sources, these integrated models are crucial for identifying optimal treatments and creating personalized genomic records (42). **Figure 4** contrasts conventional Western-derived stratification with a proposed ancestry-aware integrated framework that incorporates germline architecture, somatic spectrum, epigenetic configuration, immune microenvironment, and pharmacogenomic variants to inform personalized therapy selection. This framework represents a conceptual proposal requiring prospective development, calibration, and validation in MENA cohorts, and is not a validated or clinically implemented scoring system.

## 6 Clinical implications

### 6.1 Actionable mutation landscape

Genomic profiling in the MENA region has now reached a scale that allows meaningful clinical interpretation. A recent systematic review pooled 44 studies from 13 countries, covering over 2,500 breast cancer patients. *TP53* was the most frequently mutated gene at around 24%, followed by *PIK3CA* at roughly 10%. Close to 40% of detected variants were pathogenic or likely pathogenic.

Notably, all 11 *PIK3CA* hotspot mutations known to predict response to alpelisib were present across the dataset (12). This provides a practical basis for *PIK3CA* testing in hormone-receptor-positive, HER2-negative advanced or metastatic disease where PI3K-pathway-targeted therapy is under consideration, rather than as a universal screening strategy.

(**Figure 1**). These proportions describe the distribution of curated mutation calls rather than pooled per-patient prevalence, and no confidence intervals are reported, as the source dataset was synthesized narratively rather than through meta-analysis. **Figure 1** summarizes this distribution across gene representation, clinical classification, and variant consequence.

Saudi-specific data align with these broader trends. Whole-exome sequencing identified *PIK3CA*, *BRCA2*, and *BRCA1* among the most mutated genes, and *BRCA2* status independently predicted poorer survival (37). Founder BRCA mutations have also been reported in Lebanese (54) and Jordanian families (55). Together, these findings support the case for routine germline testing in Arab populations. However, mutations alone do not explain the full biology. How a tumor behaves also depends on the patient’s ancestral background, and this is where MENA data are still limited.

### 6.2 Ancestry and the immune microenvironment

Studies in admixed populations show that ancestry shapes breast cancer biology at a deep level. In one TCGA-based analysis, gene expression and methylation patterns shifted with ancestral proportion even within the same molecular subtype. Patients with greater African ancestry had worse 10-year survival after adjusting for stage (56). A larger study of over 13,000 tumors across six ancestry groups identified three immune phenotypes: Hot, Moderate, and Cold. Their distribution differed by ancestry, subtype, and outcome (57). Tumors from patients of African descent showed higher immune activation and BTLA-pathway signaling, while European and East Asian-ancestry tumors tended toward stromal-dominant profiles linked to poorer prognosis. These patterns were confirmed by spatial proteomics and immunohistochemistry.

Comparable ancestry-driven differences have been documented in African American (58) and Mexican Hispanic cohorts (59). If ancestry influences tumor biology, this is consistent across other populations; there is good reason to expect similar effects in Arab patients. But the data to test this are still largely missing from the MENA literature.

### 6.3 Pharmacogenomics and treatment outcomes

Population-specific variation also affects how patients metabolize drugs. Among 220 Arab breast cancer patients genotyped for *CYP2D6* and *DPYD*, about 6% carried variants with direct prescribing implications. Over 20% had variants of uncertain significance, and four *CYP2D6* alleles were entirely novel (60). A systematic review across 11 Arab countries confirmed that *CYP2D6* allele frequencies differ markedly even between neighboring nations, with CYP2D6*41 as the most common decreased-function allele in the region (61).

When targeted treatments are available, outcomes are encouraging. First-line trastuzumab, pertuzumab, and docetaxel in 75 Saudi women with HER2-positive metastatic disease yielded a 75% response rate and five-year survival above 72% (62). These results match global benchmarks. The problem is access, not efficacy. Drug availability, molecular testing capacity, and multidisciplinary care remain limited across much of the region (63), and regional survival data show only modest gains over the past decade (64).

### 6.4 Barriers to translation

Several structural issues slow the path from discovery to patient care. Sample sizes are small. The largest MENA synthesis drew on 44 separate publications to assemble 2,500 patients, far fewer than what single Western programs routinely generate (12, 65). Biobanking practices vary widely, and few centers maintain matched tumor-normal collections with standardized processing, which limits multi-site comparison (12, 66).

In Saudi Arabia, hereditary mutations in the BRCA genes remain clinically significant. In a large single-center experience involving 2,535 breast cancer patients from the Eastern Region, only 3.7% underwent BRCA testing; testing was presumably concentrated in patients meeting clinical indications such as younger age or family history, though the source study does not fully specify referral criteria (67). Of this tested subgroup, 37.5% carried a pathogenic BRCA variant - a testing-subgroup positivity rate, not a population-level prevalence estimate - illustrating both a substantial mutation burden among referred patients and a major gap in testing coverage overall. Still, cascade testing for family members is not yet standard. More broadly, despite substantial national investment in genomic infrastructure, translating research findings into routine clinical use has been slow (68). Profiling methods that have been shown to work in research settings have not been widely adopted in hospitals (37). **Table 3** provides a precision oncology readiness scorecard, mapping each domain to reported data and including a gap severity assessment.

**Table 3:** Precision oncology readiness scorecard: testing coverage, pharmacogenomics, treatment outcomes, and data infrastructure in MENA.

Domain	Sub-domain	Reported data/finding	Gap description	Severity	Ref.
Genetic testing coverage					
Genetic Testing	VUS burden	23.26% of MENA variants are VUS (12), driven by the absence of MENA data in gnomAD/ClinVar (48).	High VUS rates paralyze clinical decision-making.	HIGH	(12, 48)
Genetic Testing	Panel breadth	Western panels miss *PALB2* c.3425delT (19), *BRCA1* founders (50), stop-gain VUS (40, 42).	Actionable mutations are missed in routine practice; ancestry-aware NGS panels are essential.	HIGH	(19, 40)
Genetic Testing	BRCA testing rate	3.7% of 2,535 patients tested; 37.5% positivity among tested (67).	Massive undertesting against high mutation burden; cascade testing is not standard.	HIGH	(67)
Pharmacogenomics					
Pharmacogenomics	*CYP2D6*/*DPYD*	~6% of 220 Arab BC patients carry actionable *CYP2D6*/*DPYD* variants; >20% VUS; 4 novel	Tamoxifen/fluoropyrimidine dosing based on Western guidelines may be suboptimal or toxic.	HIGH	(60)
		*CYP2D6* alleles absent from PharmVar (60).			
Pharmacogenomics	Inter-country variation	CYP2D6*41 is the most common reduced-function allele across 11 Arab countries; marked inter-country variation (61).	Single pan-Arab reference insufficient; country-level data and CPIC adaptation required.	MOD.	(61)
Treatment access and outcomes					
Treatment	HER2+ targeted therapy	ORR 75%; 5-yr OS >72% with trastuzumab + pertuzumab + docetaxel in 75 Saudi HER2+ patients (62). Matches global benchmarks.	Efficacy is equivalent to global standards when treatment is accessible; access is the gap.	LOW	(62)
Treatment	Regional survival	Only modest survival gains in the Eastern Mediterranean region (64); BC onset ~10 yrs earlier than Western populations (63).	Late-stage presentation and limited screening drive a survival gap.	HIGH	(63, 64)
Data infrastructure					
Data Infrastructure	Database representation	MENA is severely underrepresented in TCGA, gnomAD, and GWAS Catalog (3, 12, 66).	Every clinical tool underperforms in MENA patients due to training bias.	HIGH	(3, 12, 66)
Data Infrastructure	Biobanking	Few MENA centers maintain matched tumor-normal collections (66); cascade testing	Multi-site comparison and federated models are impossible without harmonized collections.	HIGH	(66–68)
		is not standard (67); translation to clinical use is slow (68).			

(Gap severity was assigned based on (1): documented patient impact reported in the cited source (2), availability of MENA-specific data versus reliance on non-MENA benchmarks, and (3) existence of an implementable clinical pathway; categories reflect author consensus rather than a quantitative statistical threshold**.).**

Gap severity: HIGH (red) = critical deficit with documented patient impact; MODERATE (amber) = identified gap with partial solutions; LOW (green) = gap exists but non-inferior to global benchmarks. Abbreviations: VUS, variant of uncertain significance; ORR, objective response rate; OS, overall survival; CPIC, Clinical Pharmacogenetics Implementation Consortium.

## 7 Future directions

The challenges outlined above are real, but they also define a clear set of priorities. What follows are concrete steps that could meaningfully advance breast cancer care in the region.

### 7.1 A federated regional consortium

The most impactful step would be establishing a MENA Breast Cancer Genomics Consortium. A federated design in which each country retains control of its own data while sharing harmonized gene panels, analysis pipelines, and a common variant database would address the sample-size problem without the data-sovereignty issues that have blocked earlier efforts (12, 66, 68). African cancer genomics networks have already shown that federated models work well across resource-diverse settings (69), and published frameworks for structuring international oncology collaborations exist to guide the process (70). Realizing this model will require navigating substantial heterogeneity in funding capacity and data-governance frameworks across the region: GCC states with established national genomics initiatives may serve as early anchor sites, while broader MENA participation will depend on harmonizing data-sharing agreements and securing sustained funding in countries with more constrained research infrastructure.

### 7.2 Deploying emerging technologies on MENA populations

The tools to fill the region’s key knowledge gaps already exist. Single-cell RNA sequencing could uncover tumor cell states or drug resistance patterns that bulk profiling misses (71). Spatial transcriptomics could map the immune-tumor interactions underlying ancestry-linked phenotypes observed in multi-ethnic cohorts and test whether Arab patients exhibit similar patterns (57, 72).

Federated learning could train predictive models across distributed MENA datasets without requiring sensitive genomic data to leave each institution (12).

### 7.3 Early-onset biology and multi-omics integration

Breast cancer in the MENA region tends to appear around a decade earlier than in Western populations, and a large proportion of patients are diagnosed before age 50 (50, 63, 64). Whether this earlier onset is accompanied by a distinct age-stratified mutational or molecular subtype profile, as described in Western cohorts, remains largely unexamined in MENA populations and represents a priority area for future age-stratified regional sequencing studies. The reason for this earlier onset remains unclear. Ancestry-linked molecular signatures that persist within the same subtype and correlate with survival suggest that ancestry-informed classifiers could improve prognostic accuracy for younger, non-European patients (56).

Beyond its molecular implications, the younger age distribution of breast cancer in the MENA region also has direct clinical management implications. A greater proportion of patients are diagnosed during premenopausal and active life stages, requiring consideration of age-specific treatment planning, fertility and reproductive concerns, and long-term survivorship needs. These clinical considerations should be integrated into regional care pathways alongside efforts to better define the molecular basis of early-onset disease (45, 66).

Multi-omics approaches that combine gene expression, methylation, miRNA, and protein data have proven useful for risk stratification in other populations (37, 71). Still, these models were trained on predominantly European-ancestry cohorts. They need to be retested and recalibrated on MENA data. Regional centers that already have molecular profiling capacity could serve as natural pilot sites for this work (68, 70, 73).

### 7.4 Pharmacogenomic implementation and workforce development

A practical first step is a phased implementation of pharmacogenomic testing, beginning with *CYP2D6* testing for patients initiating adjuvant tamoxifen, followed by *DPYD* testing for patients being considered for fluoropyrimidine-containing regimens, as these biomarkers have distinct clinical indications and implementation requirements. *CYP2D6* genotyping informs tamoxifen dosing in patients receiving adjuvant endocrine therapy, with CPIC guidelines providing activity score-based dosing recommendations, although clinical implementation remains inconsistent even in Western health systems. In contrast, *DPYD* genotyping is relevant only for patients being considered for fluoropyrimidine-containing regimens, such as capecitabine, where CPIC guidelines recommend dose reduction or avoidance in carriers of reduced-function alleles to minimize severe toxicity. Accordingly, the initial implementation phase should prioritize *CYP2D6* testing at tertiary centers with existing genotyping capacity. A subsequent phase could expand testing to include *DPYD* as pharmacogenomic workflows and clinical expertise become established. Although most regional hospitals already possess the laboratory infrastructure to perform both assays (61), wider implementation beyond tertiary centers will require decentralization of genotyping platforms together with training of clinical pharmacists and genetic counselors, both of which remain limited outside major academic hospitals in the region (66, 68). As more regional allele-frequency data become available and Arab-specific variants are functionally characterized, testing panels can expand (60). Working with international bodies like CPIC would help adapt prescribing guidelines to local genetic profiles. None of this will happen without investing in people. Shortages in bioinformatics, genetic counselling, and clinical genomics remain the most persistent barrier across the region (66, 68). Sustained training programs, not occasional workshops, will determine whether these advances reach patients (69). **Table 4** maps each evidence base to a specific action, categorized by translational readiness tier.

**Table 4:** Evidence-to-action matrix: translating MENA breast cancer genomics into clinical practice.

Category	Evidence base	Reported finding supporting action	Recommended action	Readiness	Ref.
Germline testing					
Germline Testing	MENA-specific founders	*PALB2* c.3425delT (19); *BRCA1* c.4136_4137delCT, c.5530delC, c.4524G>A (42, 50) absent from standard panels.	Design MENA-specific NGS panels incorporating confirmed founder variants.	READY NOW	(19, 42, 50)
Germline Testing	BRCA testing rate vs. positivity	3.7% tested; 37.5% positive (67). Three Saudi *BRCA1* founders are absent from Western panels (50).	Expand to all patients ≤50 yr and all TNBC; establish cascade testing protocols.	READY NOW	(50, 67)
Somatic profiling for targeted therapy					
Somatic Profiling	Epigenetic BRCAness	*BRCA1* promoter hypermethylation creates HRD in germline-WT Saudi TNBC (10).	Include HRD and *BRCA1* methylation profiling in TNBC workup to identify PARPi-sensitive patients.	EMERGING	(10)
Somatic Profiling	*PIK3CA* for alpelisib	All 11 alpelisib-predictive *PIK3CA* hotspots are present (12); *PIK3CA*: 12.9% in the Saudi panel (37). FDA-approved for HR+/HER2− advanced BC.	Implement *PIK3CA* somatic testing as standard of care for HR+/HER2− advanced BC.	READY NOW	(12, 37)
Somatic Profiling	dMMR/MSI for pembrolizumab	Saudi panel: MMR gene mutations ~14% combined (37); signal for dMMR pembrolizumab-eligible subgroup (40).	Add MSI/MMR testing to advanced BC workup; establish dMMR eligibility pathway.	EMERGING	(37, 40)
Pharmacogenomics implementation					
Pharmacogenomics	*CYP2D6* + *DPYD*	~6% actionable; 4 novel alleles absent from PharmVar; CYP2D6*41 most common reduced-function allele (60, 61).	Phase in *CYP2D6* + *DPYD* pre-treatment genotyping; adapt CPIC guidelines to Arab allele frequencies.	READY NOW	(60, 61)
Immune stratification and multi-omics					
Multi-Omics	Single-cell/spatial/federated	Single-cell RNA-seq uncovers resistance states (71). Federated models are feasible across resource-diverse settings (12, 69).	Pilot single-cell/spatial transcriptomics at major MENA centers; build federated data governance.	FUTURE	(12, 57, 69)
Immune Stratification	Ancestry-linked phenotypes	Hot/Moderate/Cold phenotypes differ by ancestry in >13,000 tumors (57). Arab cohort: basal-myo enrichment trend (44).	Validate immune classification in MENA cohorts; test ancestry as a predictor of immunotherapy response.	EMERGING	(44, 57)

(Readiness tiers were assigned based on (1): strength and directness of the supporting evidence cited (2), existence of an implementable clinical pathway, and (3) infrastructure requirements; categories reflect author consensus rather than a quantitative statistical threshold).

Readiness tiers: READY NOW (green) = evidence sufficient for immediate clinical adoption; EMERGING (amber) = promising evidence requiring prospective MENA validation; FUTURE (blue) = requires infrastructure or large-scale data not yet available. All evidence was sourced from publications cited in this review.

## 8 Conclusions

Breast cancer in Saudi Arabia and the MENA region follows a different course than in Western populations. Women present younger, face higher rates of aggressive subtypes, and carry distinct germline and somatic variant profiles shaped by consanguinity and founder effects. Global databases largely exclude MENA patients, leaving many variants unclassified and precision oncology tools under-calibrated for this population.

Local research has made real progress. Sequencing studies have identified key somatic drivers, including *PIK3CA*, *BRCA1/2*, and *TP53*, and exact frequencies have now been established in Saudi cohorts. Germline founder mutations in *BRCA1* and *PALB2* have been confirmed. Pharmacogenomic profiling has identified novel Arab-specific *CYP2D6* alleles. Ancestry-linked transcriptomic clusters and CIMP-like methylation have been characterized.

Several practical steps can change this picture. Regional consortia should share harmonized data while keeping local control. Clinics need wider access to next-generation sequencing. Multi-omics models that integrate genomics, transcriptomics, and proteomics will reveal deeper biological insights. Routine pharmacogenomic testing for drugs like tamoxifen makes immediate sense. Training programs must build skills in bioinformatics and genetic counseling. With steady investment, MENA breast cancer treatment shifts from generic approaches to truly personalized strategies that improve survival and quality of life.
