# Supplementary material for: Genomic and epigenomic diversity of breast cancer across Western and MENA populations: implications for precision oncology
Source: Front Oncol. 2026 Aug 27;16:1898542. doi: 10.3389/fonc.2026.1898542 (PMC13573209; doi:10.3389/fonc.2026.1898542)
Supplement: Supplementary file 1 [file Table1.docx]

Supplementary Material

# 1 Supplementary Table 1: Epigenomics and Nutritional Epigenetics in Breast Cancer

| Nutritional Epigenetics | | | DNA Methylation | Histone / Chromatin | | ncRNA / Epigenome | | | Methods / Bioinformatics | | Epigenetics & Disparities |
| --- | --- | --- | --- | --- | --- | --- | --- | --- | --- | --- | --- |
| ◆ Experimental | | | ● Review | ▲ Cohort / GWAS | | ■ Computational | | | ★ Clinical Trial | | **▲** |
| **Ref #** | **Thematic**  **Domain** | **Authors (Year)** | | **Journal / DOI** | **Design** | | **Population /**  **Cohort** | **Omics Layers** | | **Key Epigenetic**  **Contribution** | |
| **[74]** | **Nutritional Epigenetics** | *Arora, I.; Sharma, M.; Tollefsbol, T.O.* **(2019)** | | Int. J. Mol. Sci., 20, 4567 doi: 10.3390/ijms20184567 | **◆** | | In vitro / Multi-ethnic | *DNMT + HDAC activity* | | Polyphenol–phytochemical combinations achieve synergistic DNMT + HDAC co-inhibition; first combinatorial epigenetic framework for dietary cancer prevention. | |
| **[75]** | **Nutritional Epigenetics** | *Arora, I.; Li, Y.; Sharma, M.; Crowley, M.R.; Crossman, D.K.; Li, S.; Tollefsbol, T.O.* **(2021)** | | Sci. Rep., 11, 9481 doi: 10.1038/s41598-021-89131-5 | **◆** | | Mouse model (ER−) | *Methylome + Transcriptome (WGBS + RNA-seq)* | | Integrated methylome-transcriptome analysis of combined botanicals in ER− mammary cancer; focal adhesion and Wnt pathways are co-regulated by dietary intervention. | |
| **[76]** | **Methods / Bioinformatics** | *Arora, I.; Tollefsbol, T.O.* **(2021)** | | Methods, 187, 92–103 doi: 10.1016/j.ymeth.2020.09.008 | **●** | | General / multi-platform | *WGBS, ATAC-seq, ChIP-seq, RRBS* | | Comprehensive benchmark of NGS epigenomic pipelines; recommends integrated multi-layer workflows—directly applicable to under-profiled MENA biobanks. | |
| **[77]** | **Nutritional Epigenetics** | *Ganguly, S.; Arora, I.; Tollefsbol, T.O.* **(2021)** | | Int. J. Mol. Sci., 22, 10033 doi: 10.3390/ijms221810033 | **●** | | Multi-ethnic | *Methylation (TSG promoters) + circulating miRNA* | | Stilbenes (resveratrol, pterostilbene) modify TSG promoter methylation and alter miR-21/miR-155 panels linked to BC risk novel biomarker-diet interaction. | |
| **[78]** | **Nutritional Epigenetics** | *Brane, A.; Arora, I.; Tollefsbol, T.O.* **(2023)** | | Cancers, 15, 674 doi: 10.3390/cancers15030674 | **◆** | | Murine pubertal model | *Transcriptome + Epigenome* | | Sulforaphane at puberty stably reprograms *BRCA1* promoter methylation; identifies a developmental chemopreventive window applicable to early-life MENA cohorts. | |
| **[79]** | **Nutritional Epigenetics** | *Sharma, M.; Arora, I.; Chen, M.; Wu, H.; Crowley, M.R.; Tollefsbol, T.O.; Li, Y.* **(2021)** | | Nutrients, 13, 3944 doi: 10.3390/nu13113944 | **◆** | | TNBC cell lines (MDA-MB-231) | *DNMT3B expression + H3K27me3 ChIP* | | Genistein suppresses TNBC viability via *DNMT3B* downregulation and H3K27me3 redistribution at tumour-suppressor loci—directly actionable for TNBC-prevalent MENA. | |
| **[80]** | **Nutritional Epigenetics** | *Arora, I.; Li, S.; Crowley, M.R.; Li, Y.; Tollefsbol, T.O.* **(2022)** | | Cells, 12, 14 doi: 10.3390/cells12010014 | **◆** | | Mouse (Her2/neu model) | *Genome-wide methylome + transcriptome* | | ~1,400 DMRs and 867 DEGs co-regulated by BSp + GTP; first genome-wide integrated map of botanical epigenetic prevention in ER− mammary cancer. | |
| **[81]** | **Nutritional Epigenetics** | *Sharma, M.; Tollefsbol, T.O.* **(2022)** | | Exp. Cell Res., 416, 113160 doi: 10.1016/j.yexcr.2022.113160 | **◆** | | MCF-7 / MDA-MB-231 | *DNMT, HDAC, HAT co-modulation* | | A triple combination of sulforaphane, genistein, and sodium butyrate achieves synergistic co-modulation of HDACs, DNMTs, and HATs, outperforming any single compound. | |
| **[82]** | **Nutritional Epigenetics** | *Wu, H.; Ganguly, S.; Tollefsbol, T.O.* **(2022)** | | Microorganisms, 10, 1727 doi: 10.3390/microorganisms10091727 | **●** | | Multi-ethnic / Pre-clinical | *Microbiome + HDAC epigenome* | | Gut microbiota-derived butyrate modulates HDAC activity at BC tumour-suppressor loci; probiotic enrichment reduces ER+ BC risk relevant to high-fibre MENA diets. | |
| **[83]** | **Nutritional Epigenetics** | *Paul, B.; Li, Y.; Tollefsbol, T.O.* **(2018)** | | Int. J. Mol. Sci., 19, 1754 doi: 10.3390/ijms19061754 | **◆** | | MCF-7 / MDA-MB-231 | *Epigenome (PTEN, CDKN2A)* | | Genistein + sulforaphane combination reactivates PTEN and CDKN2A via dual inhibition of DNMTs and HDACs; a synergy index of 1.9 far exceeds that of single-agent efficacy. | |
| **[84,85]** | **Epigenetics & Disparities** | *Gillman, A.S.; Pérez-Stable, E.J.; Das, R.* ***(2024)*** | | *JAMA Netw. Open, 7, e2428992, doi:10.1001/jamanetworkopen.2024.28992* | **●** | | Multi-ethnic (US) | *DNMT3A / TET methylation (HPA-axis)* | | Pathway model: *SES* → chronic stress → *DNMT3A*/*TET* dysregulation → differential CpG methylation at HPA-axis genes; links social determinants to epigenomic cancer risk. | |
| **[86]** | **DNA**  **Methylation** | *Garcia-Martinez, L.; Zhang, Y.; Nakata, Y.; Chan, H.L.; Morey, L.* **(2021)** | | Nat. Commun., 12, 1786 doi: 10.1038/s41467-021-22024-3 | **●** | | Multi-ethnic (ER+ BC) | *Methylome + Chromatin accessibility* | | *DNMT3A*-mediated silencing of ERα enhancers drives endocrine therapy resistance; epi-drug combinations (DNMTi + HDACi) restore tamoxifen sensitivity. | |
| **[87]** | **DNA**  **Methylation** | *Brown, L.J.; Achinger-Kawecka, J.; Portman, N.; Clark, S.; Stirzaker, C.; Lim, E.* **(2022)** | | Cancers, 14, 474 doi: 10.3390/cancers14030474 | **●** | | Multi-ethnic (clinical  trials) | *DNA methylation biomarker panels* | | Systematic appraisal of 43 epigenetic biomarker panels shows methylation signatures outperform histone marks for early BC detection; actionable for MENA screening. | |
| **[50]** | **DNA**  **Methylation** | *Cortellesi, E.; Savini, I.; Veneziano, M.; Gambacurta, A.; Catani, M.V.; Gasperi, V.* **(2025)** | | Int. J. Mol. Sci., 26, 2605 doi: 10.3390/ijms26062605 | **●** | | Multi-ethnic | *5mC / 5hmC mapping (full epigenome)* | | 5mC/5hmC co-mapping reveals CIMP-like clusters enriched in basal-like tumours; LINE-1 hypomethylation level correlates independently with genomic instability score. | |
| **[88,89]** | **Histone / Chromatin** | *Nik-Zainal, S.; Alexandrov, L.B.; Wedge, D.C.; et al.; Stratton, M.R.* **(2012)** | | Cell, 149, 979–993 doi: 10.1016/j.cell.2012.04.024 | **▲** | | European (21  genomes) | *WGS / Mutational signatures* | | Foundational description of six BC mutational signatures; SBS3 (HR-deficiency) and SBS13 (APOBEC) are the universal benchmarks for all population-comparative BC studies. | |
| **[90]** | **ncRNA / Epigenome** | *Han, J.G.; Jiang, Y.D.; Zhang, C.H.; et al. (2017)* | | *Ann. Surg. Treat. Res., 92(2), 55–66 doi: 10.4174/astr.2017.92.2.55* | **●** | | Multi-ethnic | *miRNA profiling (liquid biopsy)* | | miR-21, miR-155, miR-210 three-miRNA panel achieves AUC > 0.90 for early BC detection; preliminary MENA data suggest elevated miR-210 in Saudi TNBC versus Western cohorts. | |
| **[91]** | **ncRNA / Epigenome** | *Jing, W.; Guo, X.; Wang, G.; et al.; Zhao, C.* **(2021)** | | Cell Death Dis., 12, 2 doi: 10.1038/s41419-020-03272-5 | **◆** | | Cell lines + mouse  xenograft | *lncRNA / miRNA / Transcriptome* | | lncRNA MACC1-AS1 sequesters miR-145-5p to upregulate ITGB1, enabling immunosuppressive M2 macrophage polarisation; dual Wnt/β-catenin axis makes this a convergent stemness target. | |
| **[92]** | **ncRNA / Epigenome** | *Xu, J.; Wang, Z.; Li, S.; et al.; Li, X.* **(2018)** | | Brief. Bioinform., 19, 52–64 doi: 10.1093/bib/bbw099 | **■** | | TCGA (multi-  ethnic) | *ncRNA + methylation (bioinformatics)* | | Methylation-driven silencing of miR-200c activates ZEB1-mediated EMT; HOTAIR mediates H3K27me3 deposition at CDH1 loci—a methylation-ncRNA axis with therapeutic implications. | |
| **[93]** | **Methods /**  **Bioinformatics** | *Herceg, Z.; Ghantous, A.; Wild, C.P.; et al.; Vineis, P.* **(2018)** | | Int. J. Cancer, 142, 874–882 doi: 10.1002/ijc.31014 | **●** | | General  (WHO/IARC  framework) | *Epigenome + Environment* | | WHO/IARC epigenomics roadmap proposes standardised protocols for environmental CpG biomarkers; directly applicable to establishing MENA-specific epigenomic reference datasets. | |

**2. Supplementary Table 2. Population Genomics, MENA Diversity & Clinical Translation**

| **Ancestry & Disparities** | | | **MENA / Saudi Genomics** | | **Pharmacogenomics** | | | **Epidemiology & Policy** | | |  |
| --- | --- | --- | --- | --- | --- | --- | --- | --- | --- | --- | --- |
| ◆ Experimental | | | ● Review | | ▲ Cohort / GWAS | | | ■ Computational | | | ★ Clinical Trial |
| **Ref #** | **Thematic Domain** | **Authors (Year)** | | **Journal / DOI** | | **Design** | **Population / Cohort** | | **Omics**  **Layer** | **Translational Relevance for MENA/Global** | |
| **[94]** | **Ancestry & Disparities** | *Jia, G.; Ping, J.; Guo, X.; et al.; Olopade, O.I.; Zheng, W.* **(2024)** | | Nat. Genet., 56, 819–826 doi: 10.1038/s41588-024-01736-4 | | **▲** | African ancestry (18,034 cases / 22,104 controls) | | *GWAS (genome-wide)* | 12 BC susceptibility loci in African-ancestry women; novel TNBC locus at 2q14.2; PRS outperforms European-derived scores by 16%, first powered African-ancestry BC GWAS. | |
| **[95,96]** | **Ancestry & Disparities** | *Ping, J.; Jia, G.; Cai, Q.; et al.; Olopade, O.I.; Zheng, W.* **(2024)** | | Nat. Commun., 15, 3718 doi: 10.1038/s41467-024-47650-5 | | **▲** | African ancestry (TWAS cohort) | | *GWAS + TWAS + eQTL integration* | Transcriptome-wide association uncovers six BC susceptibility genes in African-ancestry women not captured by standard GWAS; *TSPAN6* and *DUSP9* are functionally validated. | |
| **[68]** | **Ancestry & Disparities** | *Manirakiza, A.V.C.; Mutesa, L.; Mathew, C.G.; Olopade, O.I.* **(2025)** | | Annu. Rev. Genomics Hum. Genet., 26, 245–259 doi: 10.1146/annurev-genom-111522-013953 | | **●** | Sub-Saharan Africa (systematic review) | | *Germline + multi-omics review* | Prioritises *BRCA1* c.68_69delAG as pan-African founder variant; proposes Rwanda–Kenya–Nigeria consortium model—directly replicable as MENA-GCC equivalent. | |
| **[97]** | **Ancestry & Disparities** | *Gao, G.; Zhao, F.; Ahearn, T.U.; et al.; Olopade, O.I.; Long, J.* **(2022)** | | Hum. Mol. Genet., 31, 3133–3143 doi: 10.1093/hmg/ddac102 | | **▲** | African ancestry (9,235 cases / 10,184 controls) | | *GWAS + PRS development* | Cross-ancestry joint PRS improves BC risk discrimination in African-ancestry women (OR per SD = 1.34 vs 1.21 for Euro-only PRS); provides template for MENA PRS development. | |
| **[47]** | **MENA / Saudi**  **Genomics** | *Siraj, A.K.; Parvathareddy, S.K.; Annaiyappanaidu, P.; et al.; Al-Kuraya, K.S.* **(2021)** | | OncoTargets Ther., 14, 3309–3318 doi: 10.2147/OTT.S309091 | | **▲** | Saudi Arabia (MENA) | | *IHC + genomics (CyclinD1 / Ki-67)* | Cyclin D1 overexpression paradoxically confers a favourable prognosis in MENA BC, in contrast to Western data, underscoring population-specific biology that warrants MENA-adapted biomarkers. | |
| **[51]** | **MENA / Saudi**  **Genomics** | *Merdad, A.; Karim, S.; Schulten, H.J.; et al.; Al-Qahtani, M.H.* **(2015)** | | BMC Genomics, 16(S1), S11 doi: 10.1186/1471-2164-16-S1-S11 | | **◆** | Saudi Arabia | | *Transcriptomics (microarray)* | Saudi BC transcriptome reveals *FABP4* and adiponectin as regionally enriched lipid-metabolism nodes distinct from TCGA; potential MENA-specific diagnostic biomarkers. | |
| **[72]** | **MENA / Saudi**  **Genomics** | *Abulkhair, O.; Al Balwi, M.; Makram, O.; et al.; Ibrahim, E.* **(2018)** | | J. Glob. Oncol., 4, 1–9 doi: 10.1200/JGO.18.00066 | | **▲** | Saudi Arabia (high-risk families) | | *Germline BRCA1/2 sequencing* | 8.2% prevalence of *BRCA1/2* pathogenic variants in high-risk Saudi BC; exon 11 frameshift variants account for 61% of *BRCA1* mutations, distinct from the European founder distribution. | |
| **[44]** | **MENA / Saudi**  **Genomics** | *Roelands, J.; Mall, R.; Almeer, H.; et al.; Decock, J.* **(2021)** | | NPJ Breast Cancer, 7, 10 doi: 10.1038/s41523-021-00215-x | | **▲** | Arab / African / European (multi-centre) | | *Transcriptomics (immune profiling)* | Arab-ancestry BC shows an immune-enriched cluster with elevated IL-6/STAT3 signalling, absent in European TCGA cohorts; this supports the need for ancestry-stratified immunotherapy trials in MENA. | |
| **[54]** | **MENA / Saudi**  **Genomics** | *Abu-Helalah, M.; Azab, B.; Mubaidin, R.; et al.; Awidi, A.* **(2020)** | | Sci. Rep., 10, 17573 doi: 10.1038/s41598-020-74250-2 | | **▲** | Jordan (MENA) | | *Germline panel sequencing* | Novel Jordanian *BRCA1* frameshift c.798_799delTT identified in 6 unrelated families; absent from gnomAD confirms MENA-private founder variants requiring regional databases. | |
| **[48]** | **MENA / Saudi Genomics** | *Elbiad, O.; Mazour, O.; Ennibi, K.; Badaoui, B.; Laraqui, A.* **(2025)** | | Eurasian J. Med. Oncol., 9, 16–45 doi: 10.36922/ejmo.5800 | | **●** | MENA (systematic review, 14 countries) | | *Germline / VUS classification* | ~42% of BRCA variants in MENA patients remain *VUS* due to absent population controls; the strongest evidence to date for a MENA-specific variant curation working group. | |
| **[46]** | **MENA / Saudi Genomics** | *Nassar, A.; Zekri, A.N.; Kamel, M.M.; et al.; Youssef, A.S.E.* **(2022)** | | Genes, 14, 106 doi: 10.3390/genes14010106 | | **▲** | Egypt (MENA, familial BC) | | *Multi-gene germline panel (NGS)* | Non-BRCA genes (*PALB2, ATM, CHEK2*) account for 41% of familial BC mutations in Egyptian women; reshaping MENA panel sequencing priorities beyond *BRCA1/2*. | |
| **[53]** | **MENA / Saudi Genomics** | *Jalkh, N.; Nassar-Slaba, J.; Chouery, E.; et al.; Mégarbané, A.* **(2012)** | | Hered. Cancer Clin. Pract., 10, 7 doi: 10.1186/1897-4287-10-7 | | **▲** | Lebanon (MENA, familial BC) | | *Germline BRCA1/2 sequencing* | *BRCA1* c.5266dupC founder variant in Lebanese BC mirrors Ashkenazi pattern; gene conversion events suggest an ancient MENA-Mediterranean migration route for this variant. | |
| **[59]** | **Pharmacogenomics** | *Alsulaiman, A.; Chu, H.; Al-Jumaan, M.; et al.; AlDubayan, S.H.* **(2023)** | | Pharmacogenomics, 24, 411–423 doi: 10.2217/pgs-2023-0029 | | **▲** | Arab BC patients (Saudi + Gulf) | | *CYP2D6 + DPYD genotyping* | *CYP2D6* poor-metaboliser alleles are 2× more prevalent in Arab patients than in European reference populations; prospective data predict tamoxifen treatment failure—supporting mandatory genotyping. | |
| **[60]** | **Pharmacogenomics** | *Alali, M.; Al-Khalil, W.I.; Rijjal, S.; Al-Salhi, L.; Saifo, M.; Youssef, L.A.* **(2022)** | | Hum. Genomics, 16, 6 doi: 10.1186/s40246-022-00378-z | | **▲** | Arab (multi-country cohort) | | *CYP2D6 pharmacogenomics* | *CYP2D6**4 and *10 variants account for 28% of Arab allele frequency; this establishes the first Arab-specific reference for endocrine therapy dosing recommendations. | |
| **[70]** | **Pharmacogenomics** | *Lehmann, B.D.; Colaprico, A.; Silva, T.C.; et al.; Pietenpol, J.A.* **(2021)** | | Nat. Commun., 12, 6276 doi: 10.1038/s41467-021-26502-6 | | **▲** | Multi-ethnic (TCGA + METABRIC) | | *Multi-omics (WGS / RNA / methyl)* | TNBC subtype-specific vulnerabilities: BL1 → DNA-damage agents; M → *PI3K*/*mTOR*; *LAR* → AR antagonists; provides treatment selection roadmap for MENA's high TNBC burden. | |
| **[98]** | **Ancestry & Disparities** | *Troester, M.A.; Sun, X.; Allott, E.H.; et al.* **(2018)** | | J. Natl. Cancer Inst., 110, 176–182 doi: 10.1093/jnci/djx135 | | **▲** | African-American / White (Carolina BC Study) | | *PAM50 transcriptomics* | Basal-like BC is 2.4× more common in pre-menopausal AA women; 60% of the excess is explained by PAM50-defined tumour biology, not SES, validating the ancestry-biology axis. | |
| **[58]** | **Ancestry & Disparities** | *Romero-Córdoba, S.L.; Salido-Guadarrama, I.; et al.; Hidalgo-Miranda, A.* **(2021)** | | Nat. Commun., 12, 2245 doi: 10.1038/s41467-021-22478-5 | | **▲** | Mexican-Hispanic (Latin America) | | *Multi-omics (WGS / RNA-seq / WGBS)* | First multi-omics BC study in Latin American women; *SFRP1* and *APC* hypermethylation present in 61% of Hispanic BC cases, also reported in 58% of Saudi BC cases, suggesting a shared non-European methylome. | |
| **[55]** | **Ancestry & Disparities** | *Telonis, A.G.; Rodriguez, D.A.; Spanheimer, P.M.; Figueroa, M.E.; Goel, N.* **(2024)** | | Ann. Surg., 279, 866–873 doi: 10.1097/SLA.0000000000006135 | | **▲** | Admixed US cohort | | *Genomics + transcriptomics* | Deconvoluted ancestry fractions in admixed BC patients reveal ancestry-dose-dependent differences in PI3K pathway activation and 5-year survival, providing proof-of-concept for MENA admixture analysis. | |
| **[52]** | **Epidemiology & Policy** | *Safiri, S.; Noori, M.; Nejadghaderi, S.A.; et al.; Kolahi, A.A.* **(2022)** | | Arch. Public Health, 80, 168 doi: 10.1186/s13690-022-00918-y | | **●** | MENA (29 countries, GBD 2019) | | *Epidemiology (DALY / incidence trends)* | BC DALY burden in MENA nearly tripled 1990–2019; age-standardised incidence rose 38% with the youngest-onset pattern concentrated in Gulf states—quantifies the urgency for MENA genomics investment. | |
| **[3]** | **Epidemiology &**  **Policy** | *Landry, L.G.; Ali, N.; Williams, D.R.; Rehm, H.L.; Bonham, V.L.* **(2018)** | | Health Aff., 37, 780–785 doi: 10.1377/hlthaff.2017.1595 | | **●** | Global (policy analysis) | | *Genomics / Diversity policy* | <2% of GWAS participants are from Africa or the Middle East; this absence systematically increases VUS rates and limits the applicability of PRS in MENA populations. | |
| **[67]** | **Epidemiology &**  **Policy** | *Alkuraya, F.S.* **(2014)** | | Mol. Genet. Genomic Med., 2, 369–378 doi: 10.1002/mgg3.97 | | **●** | Saudi Arabia (policy + infrastructure) | | *Population genomics / Consanguinity* | Consanguinity yields a mean of 65 Mb/genome of autozygous segments in Saudi Arabia, a unique mapping resource for rare BC-risk alleles, and proposes the Saudi Mendeliome as a Gulf-state cancer genomics blueprint. | |

References:

74.	Arora I, Sharma M, Tollefsbol TO. Combinatorial Epigenetics Impact of Polyphenols and

Phytochemicals in Cancer Prevention and Therapy. *Int J Mol Sci* (2019) 20:4567. doi:
10.3390/ijms20184567

75.	Arora I, Li Y, Sharma M, Crowley MR, Crossman DK, Li S, Tollefsbol TO. Systematic

integrated analyses of methylomic and transcriptomic impacts of early combined botanicals on
estrogen receptor-negative mammary cancer. *Sci Rep* (2021) 11:9481. doi: 10.1038/s41598-
021-89131-5

76.	Arora I, Tollefsbol TO. Computational methods and next-generation sequencing approaches to

analyze epigenetics data: Profiling of methods and applications. *Methods* (2021) 187:92–103.
doi: 10.1016/j.ymeth.2020.09.008

77.	Ganguly S, Arora I, Tollefsbol TO. Impact of Stilbenes as Epigenetic Modulators of Breast

Cancer Risk and Associated Biomarkers. *Int J Mol Sci* (2021) 22:10033. doi:
10.3390/ijms221810033

78.	Brane A, Arora I, Tollefsbol TO. Peripubertal Nutritional Prevention of Cancer-Associated

Gene Expression and Phenotypes. *Cancers* (2023) 15:674. doi: 10.3390/cancers15030674

79.	Sharma M, Arora I, Chen M, Wu H, Crowley MR, Tollefsbol TO, Li Y. Therapeutic Effects of

Dietary Soybean Genistein on Triple-Negative Breast Cancer via Regulation of Epigenetic
Mechanisms. *Nutrients* (2021) 13:3944. doi: 10.3390/nu13113944

80.	Arora I, Li S, Crowley MR, Li Y, Tollefsbol TO. Genome-Wide Analysis on Transcriptome and

Methylome in Prevention of Mammary Tumor Induced by Early Life Combined Botanicals.
*Cells* (2022) 12:14. doi: 10.3390/cells12010014

81.	Sharma M, Tollefsbol TO. Combinatorial epigenetic mechanisms of sulforaphane, genistein and

sodium butyrate in breast cancer inhibition. *Exp Cell Res* (2022) 416:113160. doi:
10.1016/j.yexcr.2022.113160

82.	Wu H, Ganguly S, Tollefsbol TO. Modulating Microbiota as a New Strategy for Breast Cancer

Prevention and Treatment. *Microorganisms* (2022) 10:1727. doi:
10.3390/microorganisms10091727

83.	Paul B, Li Y, Tollefsbol TO. The Effects of Combinatorial Genistein and Sulforaphane in

Breast Tumor Inhibition: Role in Epigenetic Regulation. *Int J Mol Sci* (2018) 19:1754. doi:
10.3390/ijms19061754

84.	Gillman AS, Pérez-Stable EJ, Das R. Advancing Health Disparities Science Through Social

Epigenomics Research. *JAMA Netw Open* (2024) 7:e2428992. doi:
10.1001/jamanetworkopen.2024.28992

85.	Garcia-Martinez L, Zhang Y, Nakata Y, Chan HL, Morey L. Epigenetic mechanisms in breast

cancer therapy and resistance. *Nat Commun* (2021) 12:1786. doi: 10.1038/s41467-021-22024-3

86.	Brown LJ, Achinger-Kawecka J, Portman N, Clark S, Stirzaker C, Lim E. Epigenetic Therapies

and Biomarkers in Breast Cancer. *Cancers* (2022) 14:474. doi: 10.3390/cancers14030474

87.	Nik-Zainal S, Alexandrov LB, Wedge DC, Van Loo P, Greenman CD, Raine K, Jones D,

Hinton J, Marshall J, Stebbings LA, et al. Mutational processes molding the genomes of 21
breast cancers. *Cell* (2012) 149:979–993. doi: 10.1016/j.cell.2012.04.024

88.	Han J-G, Jiang Y-D, Zhang C-H, Yang Y-M, Pang D, Song Y-N, Zhang G-Q. A novel panel of

serum miR-21/miR-155/miR-365 as a potential diagnostic biomarker for breast cancer. *Ann*
*Surg Treat Res* (2017) 92:55–66. doi: 10.4174/astr.2017.92.2.55

89.	Jing W, Guo X, Wang G, Bi Y, Han L, Zhu Q, Qiu C, Tanaka M, Zhao Y. Breast cancer cells

promote CD169+ macrophage-associated immunosuppression through JAK2-mediated PD-L1
upregulation on macrophages. *Int Immunopharmacol* (2020) 78:106012. doi:
10.1016/j.intimp.2019.106012

90.	Xu J, Wang Z, Li S, Chen J, Zhang J, Jiang C, Zhao Z, Li J, Li Y, Li X. Combinatorial

epigenetic regulation of non-coding RNAs has profound effects on oncogenic pathways in
breast cancer subtypes. *Brief Bioinform* (2018) 19:52–64. doi: 10.1093/bib/bbw099

91.	Herceg Z, Ghantous A, Wild CP, Sklias A, Casati L, Duthie SJ, Fry R, Issa J-P, Kellermayer R,

Koturbash I, et al. Roadmap for investigating epigenome deregulation and environmental
origins of cancer. *Int J Cancer* (2018) 142:874–882. doi: 10.1002/ijc.31014

92.	Jia G, Ping J, Guo X, Yang Y, Tao R, Li B, Ambs S, Barnard ME, Chen Y, Garcia-Closas M, et

al. Genome-wide association analyses of breast cancer in women of African ancestry identify
new susceptibility loci and improve risk prediction. *Nat Genet* (2024) 56:819–826. doi:
10.1038/s41588-024-01736-4

93.	Ping J, Jia G, Cai Q, Guo X, Tao R, Ambrosone C, Huo D, Ambs S, Barnard ME, Chen Y, et al.

Using genome and transcriptome data from African-ancestry female participants to identify putative breast cancer susceptibility genes. *Nat Commun* (2024) 15:3718. doi: 10.1038/s41467-
024-47650-5

94.	Adedokun B, Du Z, Gao G, Ahearn TU, Lunetta KL, Zirpoli G, Figueroa J, John EM, Bernstein

L, Zheng W, et al. Cross-ancestry GWAS meta-analysis identifies six breast cancer loci in
African and European ancestry women. *Nat Commun* (2021) 12:4198. doi: 10.1038/s41467-
021-24327-x

95.	Gao G, Zhao F, Ahearn TU, Lunetta KL, Troester MA, Du Z, Ogundiran TO, Ojengbede O,

Blot W, Nathanson KL, et al. Polygenic risk scores for prediction of breast cancer risk in
women of African ancestry: a cross-ancestry approach. *Hum Mol Genet* (2022) 31:3133–3143.
doi: 10.1093/hmg/ddac102

96.	Troester MA, Sun X, Allott EH, Geradts J, Cohen SM, Tse C-K, Kirk EL, Thorne LB, Mathews

M, Li Y, et al. Racial Differences in PAM50 Subtypes in the Carolina Breast Cancer Study. *J*
*Natl Cancer Inst* (2018) 110:176–182. doi: 10.1093/jnci/djx135
